# Global health journals need to address equity, diversity and inclusion

**DOI:** 10.1136/bmjgh-2019-002018

**Published:** 2019-10-18

**Authors:** Vaidehi Nafade, Paulami Sen, Madhukar Pai

**Affiliations:** 1 Epidemiology & Biostatistics, McGill University, Montreal, Quebec, Canada; 2 McGill Global Health Programs, McGill University, Montreal, Quebec, Canada

**Keywords:** health policies and all other topics

**Summary box**

- Equity, diversity and inclusion are necessary in all fields of research, but these values are particularly relevant in global health.
- We examined the composition of editors and editorial board members of 12 major global health journals to examine diversity and inclusion.
- Across all journals, 35% (195 of 551) of editors were female, and 33% (184 of 551) were based in low-income and middle-income countries (LMICs). Only 11% (61 of 551) of all editors were women based in LMICs. Only 4% of the editors with leadership roles were women from LMICs.
- We make a plea for all global health journals to take a pledge for gender parity and greater inclusion of experts from the Global South.

Equity is widely accepted as the central goal of all global health endeavours.^1^ And diversity and inclusion are critical, since all practitioners of global health will readily endorse the need to abandon colonial approaches.^2^

In reality, even today, global health remains entrenched in colonial structures and power dynamics, where high-income country (HIC) experts and institutions are valued much more than expertise in low-income and middle-income countries (LMICs).^3–5^

Most global health research funds are spent in HICs,^6^ and HIC experts dominate advisory boards of major funders and global health agencies.^5^ Data show under-representation of LMIC authors on research publications that are about LMICs,^7^ and parachute research continues to be a persistent concern.^8^

Global health conferences and commissions are typically hosted in HICs,^9^ and their agendas are shaped by HIC speakers and chairs.^5^ Gender inequality is another concern, with data showing that women are underrepresented at all stages of the research and publishing process, from authorship, to peer review, to editorship.^10^

What about editorial boards of global health journals? We examined the composition of editors and editorial board members of 12 major global health journals to examine diversity and inclusion. Although global health research is published in a variety of journals, for the sake of simplicity and clarity, we focused on the subset of journals which explicitly included ‘global health’ or ‘international health’ in the journal title.

We grouped editors and editorial board members according to their leadership role and identified the primary location and gender of each person. For simplicity, countries were classified as HIC versus LMIC, according to World Bank definitions. To capture leadership and responsibility, we created three simple groups: group 1 included editors-in-chief, or those in leadership roles; group 2 included senior, deputy or associate editors, as well as editors responsible for specialist content (eg, web and social media); and group 3 included editorial board or advisory board members.

All information was initially extracted from the journal websites by one author (VN) and then cross-checked for accuracy by a second author (PS). Extracted data were then shared with the chief editor or manager of each journal to be confirmed. The final dataset included corrections sent in by the journals.

**Figure 1:**
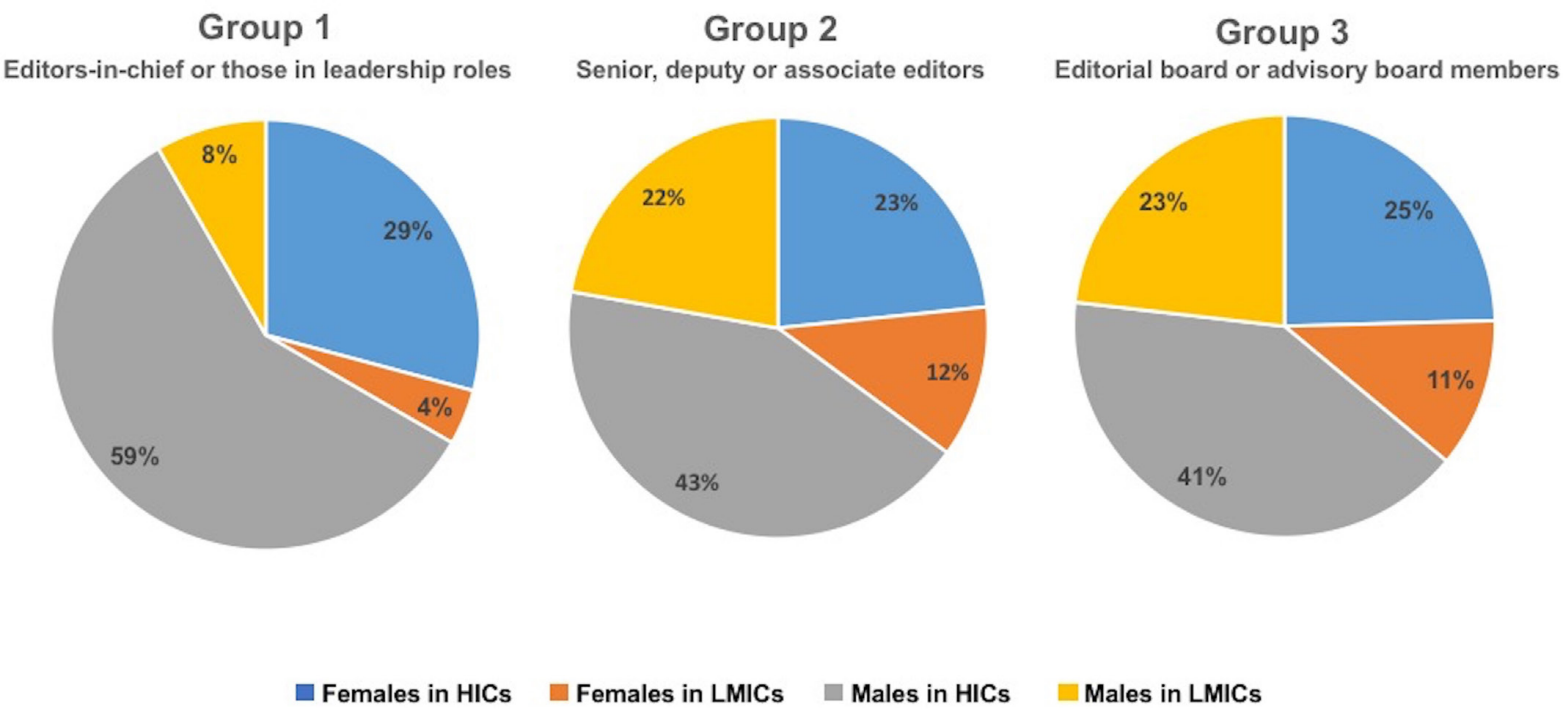
Global health editors and editorial board members according to location, gender and group. Group 1 included editors-in-chief, or those in leadership roles; group 2 included senior, deputy or associate editors; and group 3 included editorial board or advisory board members. HICs, high-income countries; LMICs, low-income and middle-income countries.

In total, the sample comprised 551 editors or editorial board members across 12 journals. Table 1 includes the breakdown of editors by location and gender for all journals, and figure 1 displays this data according to group.

**Table 1:** Global health editors and editorial board members according to location and gender

Journal	Based in LMICs			Based in HICs			Total
	Female editors	Male editors	All editors	Female editors	Male editors	All editors	
*BMJ Global Health*	5	6	11	9	15	24	35
*Clinical Epidemiology and Global Health*	19	34	53	2	10	12	65
*Global Health Action*	1	4	5	11	12	23	28
*Global Health Governance*	1	3	4	26	28	54	58
*Global Health Research and Policy*	7	28	35	9	10	19	54
*Global Health: Science and Practice*	3	3	6	11	13	24	30
*Global Public Health*	10	13	23	18	21	39	62
*International Health*	2	7	9	7	20	27	36
*Journal of Epidemiology and Global Health*	0	1	1	5	29	34	35
*Journal of Global Health*	3	8	11	19	30	49	60
*Lancet Global Health*	5	6	11	7	8	15	26
*Tropical Medicine & International Health*	5	10	15	10	37	47	62

HICs, high-income countries; LMICs, low-income and middle-income countries.

Across all journals, 35% (195 of 551) of editors were female, and 33% (184 of 551) were based in an LMIC. Only 11% (61 of 551) of all editors were women based in LMICs. Male editors in HICs were over-represented among editors-in-chief, comprising 59% (14 HIC male editors out of 24 total senior editors) of this sample compared with 42% (233 HIC male editors out of 551 total editors) of the full sample. Only one editor in group 1 (ie, editor-in-chief) was a woman from an LMIC. Among the 12 journals, all except two were managed by institutions in USA or Europe, and six of 12 were open-access.

Table 2 shows the ranking of journals, with respect to inclusion of women and experts from LMICs. *Global Health: Science and Practice* ranked the highest for inclusion of women, while *Clinical Epidemiology and Global Health* ranked the highest for inclusion of LMIC experts. The *Journal of Epidemiology and Global Health* ranked the lowest for inclusion of women, while as well as inclusion of LMIC experts.

**Table 2:** Ranking of global health journals, with respect to inclusion of women and LMIC experts in editorial boards

	% Female	Ranking	% LMIC	Ranking	% Female+LMIC	Ranking
Lancet Global Health	0.462	3	0.423	3	0.192	2
BMJ Global Health	0.400	6	0.314	5	0.143	4
Journal of Global Health	0.367	7	0.183	9	0.050	9
International Health	0.250	10	0.250	6	0.056	8
Global Health Research and Policy	0.296	9	0.648	2	0.130	5
Global Public Health	0.452	4	0.371	4	0.161	3
Global Health: Science and Practice	0.467	1	0.200	8	0.100	6
Global Health Action	0.429	5	0.179	10	0.036	10
Journal of Epidemiology and Global Health	0.143	12	0.057	12	0.029	11
Global Health Governance	0.466	2	0.069	11	0.017	12
Tropical Medicine & International Health	0.242	11	0.242	7	0.081	7
Clinical Epidemiology and Global Health	0.323	8	0.815	1	0.292	1

Equity, diversity and inclusion are necessary in all fields of research, but these values are particularly relevant in global health, as the burden of disease and disability falls disproportionately on LMICs. Experts from the Global South, therefore, have greater knowledge and lived experience about the issues involved, and can offer deeper insights into potential solutions. Without adequate representation on editorial boards, research from LMICs—where the highest burden is—may be deemed less relevant or evaluated less fairly when experts from these countries aren’t represented on editorial boards.^11^ Furthermore, researchers outside of Europe and North America may receive fewer opportunities to participate in the publishing process, which may in turn affect their professional development, ability to attract grants, and serve on policy committees.

These structural biases have been described in the context of gender equality. Our finding that women only comprise 35% of all editors confirms that global health journals suffer from the same lack of gender diversity as other scientific fields. Moreover, the finding that women in LMICs only account for 11% of all editors shows that women face overlapping systems of discrimination. This gap only worsens at higher levels of leadership, with only 4% of the editors with leadership roles being women in LMICs.

Our small study has limitations and did not aim to cover all journals that publish global health research. We also acknowledge that our simplistic categories of HIC versus LMIC do not quite capture the realities. But the data do suggest that journals that are explicitly focused on global or international health are not walking the talk to address equity and diversity.

We agree with Sheikh and colleagues who argue that ‘the Global Health community needs to be the change it wants to see in the world, and take a pledge for greater inclusivity’.^5^ We also make a plea for all global health journals to take a pledge for gender parity and greater inclusion of LMIC experts.

There is growing pushback about manels in meetings and conferences,^12^ and initiatives such as Women in Global Health (https://www.womeningh.org/) are successfully advocating for greater representation of women in all aspects of global health. This year, *The Lancet* group of journals has committed to achieving gender parity by 2020.^13^

However, without addressing inclusion of expertise from the Global South, gender parity might result in privileged women experts from HICs dominating global health.^14^ So, it is critical to also ensure that women experts from LMICs are adequately represented.

As is always the case, there are deeper layers to the problem and addressing them will require much more than the reconfiguration of editorial boards. It will require us to collectively ask and address hard questions such as, why, in 2019, most global health journals are headquartered in London, New York, or Baltimore, and run by colonial-era institutions,^15^ and what that means for equity and inclusion?
